# Crystal orientation of epitaxial film deposited on silicon surface

**DOI:** 10.1038/s41598-024-61564-8

**Published:** 2024-05-13

**Authors:** Satoru Kaneko, Takashi Tokumasu, Manabu Yasui, Masahito Kurouchi, Daishi Shiojiri, Shigeo Yasuhara, Sumanta Kumar Sahoo, Musa Mutlu Can, Ruei Sung Yu, Kripasindhu Sardar, Masahiro Yoshimura, Masaki Azuma, Akifumi Matsuda, Mamoru Yoshimoto

**Affiliations:** 1 Kanagawa Institute of Industrial Science and Technology (KISTEC), Ebina, Kanagawa 243-0435 Japan; 2 Tohoku University, Sendai, Miyagi 980-8577 Japan; 3 Japan Advanced Chemicals, Sagamihara, Kanagawa 252-0243 Japan; 4 Radhakrishna Institute of Technology and Engineering, Bhubaneswar, Odisha 752057 India; 5 Istanbul University, Istanbul, 34134 Turkey; 6 Asia University, Taichung, 41354 Taiwan; 7 National Cheng Kung University, Tainan, 701 Taiwan; 8 Tokyo Institute of Technology, Yokohama, 226-8502 Japan

**Keywords:** Surfaces, interfaces and thin films, Synthesis and processing

## Abstract

Direct growth of oxide film on silicon is usually prevented by extensive diffusion or chemical reaction between silicon (Si) and oxide materials. Thermodynamic stability of binary oxides is comprehensively investigated on Si substrates and shows possibility of chemical reaction of oxide materials on Si surface. However, the thermodynamic stability does not include any crystallographic factors, which is required for epitaxial growth. Adsorption energy evaluated by total energy estimated with the density functional theory predicted the orientation of epitaxial film growth on Si surface. For lower computing cost, the adsorption energy was estimated without any structural optimization (simple total of energy method). Although the adsorption energies were different on simple ToE method, the crystal orientation of epitaxial growth showed the same direction with/without the structural optimization. The results were agreed with previous simulations including structural optimization. Magnesium oxide (MgO), as example of epitaxial film, was experimentally deposited on Si substrates and compared with the results from the adsorption evaluation. X-ray diffraction showed cubic on cubic growth [MgO(100)//Si(100) and MgO(001)//Si(001)] which agreed with the results of the adsorption energy.

## Introduction

Direct growth of oxide film on silicon surface has been explored to combine silicon technology and functioning oxide materials for device applications^1–9^. However silicon surface can be easily oxidized in oxygen atmosphere before oxide film deposition. And interdiffusion or chemical reaction between silicon and those oxide materials prevent direct growth of oxide film on silicon surface. In general, a buffer layer is required between oxide and silicon surface for epitaxial growth^10–14^. For selection of oxide materials expected to grow on silicon surface, Schlom et al. reported thermodynamic stability of binary oxides in contact with silicon surface^15^, and comprehensively investigate the thermodynamic stability of more than 80 binary oxides. However, the thermodynamic stability is not concerned with any information about crystallography such as orientation of epitaxial film growth.

**Figure 1:**
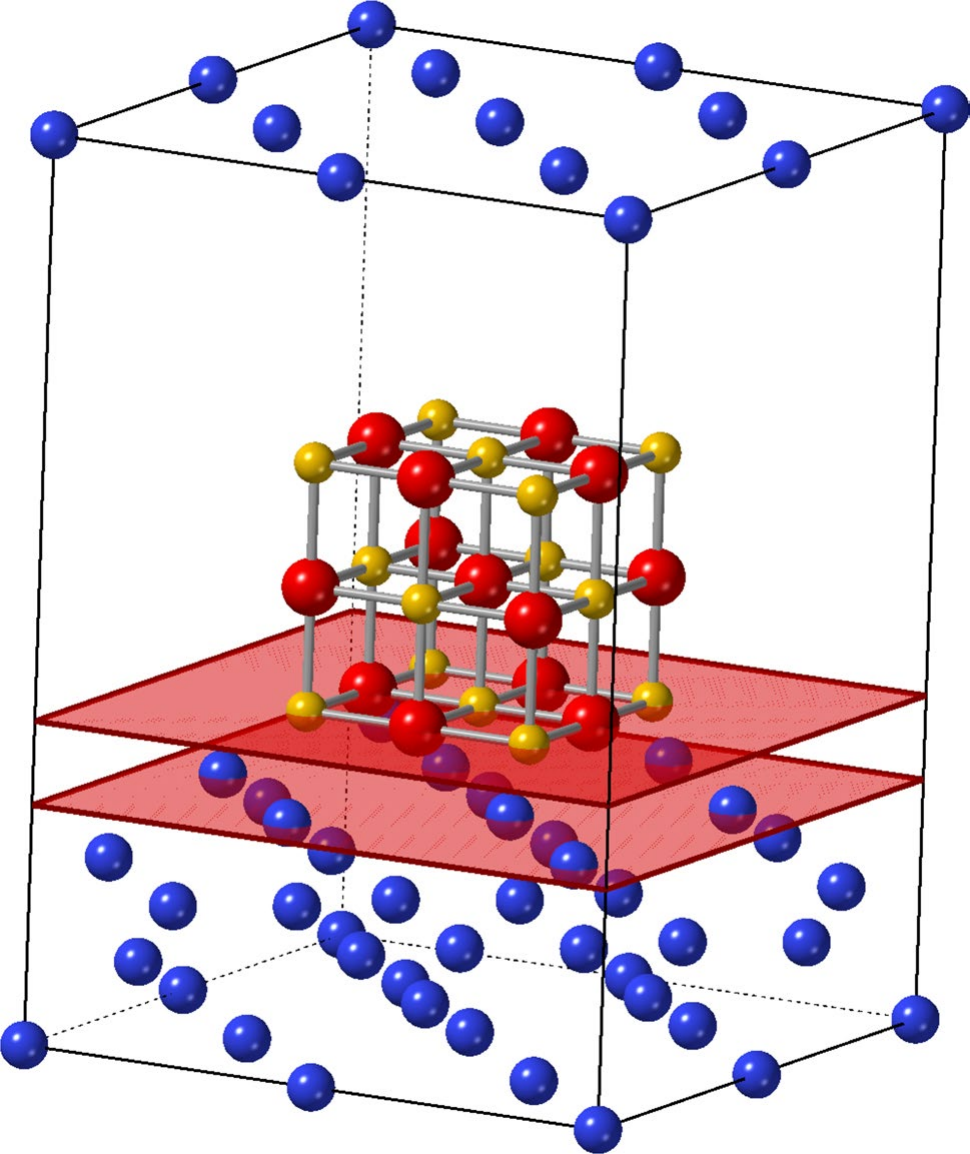
Schematics of supercell consisting MgO cluster placed on silicon surface with varied distances. The red flat planes show the surface of Si substrate and the bottom of MgO cluster.

In order to evaluate the crystallographic stability, an adsorption energy was estimated on target materials. Magnesium oxide (MgO), for an example, was placed on silicon (Si) surface (supercell) as shown in Fig. 1 and the total energy was calculated by using molecular dynamics, and the crystal orientation of epitaxial growth was evaluated by the absorption energy^16^. In previous study, carbon clusters were also placed on a variety of substrates, and the adsorption energy was used to select an appropriate substrate for graphene growth, and *super flat* graphene^17,18^ was verified on the candidate target. For graphene growth, carbon clusters were also optimized as well as optimizing substrate surface before constructing supercells, and position and orientation of clusters were also optimized during the calculation.

Optimization of substrate surface and cluster migration requires time-cost and resources. Although an accurate calculation is available with time consuming and expense, rough estimations can be enough for many cases. In this study, stability of crystal orientation of MgO film was evaluated on Si surface. Many study has been explored on epitaxial MgO on Si substrate^2–4,6–8,19–26^, however some difficulty still remain on the topic. In addition to aforementioned thermal stability, the difficulty of epitaxial growth comes from large lattice mismatch and narrow deposition conditions^27^, and post-annealing or buffer layer might be required^13^. The lattice constants, for example, of MgO is 4.21 and whereas that of Si is 5.431Å, implying a mismatch of 22%, which can be more than enough reason to exclude MgO from candidate materials deposited on Si substrate. However, epitaxial growth of MgO has been reported on Si(001) substrates^3,4,7,8^.

**Figure 2:**
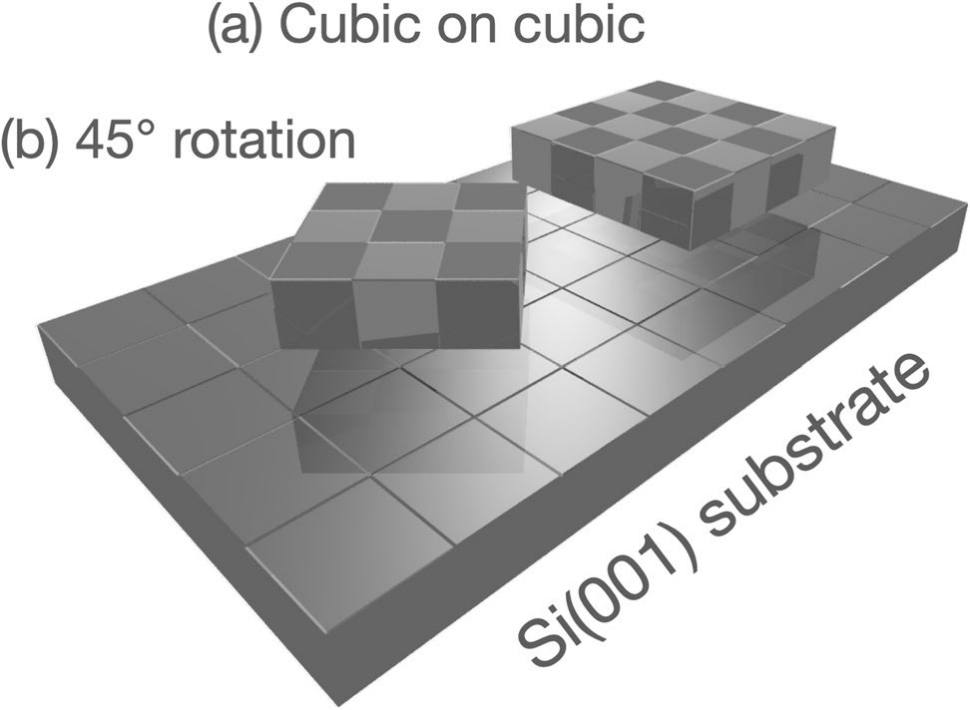
Schematics of growth of MgO deposited on Si substrate. MgO(001) cluster grows on Si(001) along with the relation of (**a**) cubic on cubic growth [MgO(100) // Si(100)] and (**b**) 45^∘^ rotation growth [MgO(110) // Si(100)].

In general, MgO can epitaxially grow on Si(001) substrate with the relation of cubic on cubic growth [MgO(001) // Si(001) and MgO(100) // Si(100)] or 45^∘^ rotation growth [MgO(001) // Si(001) and MgO(110) // Si(100)], as shown in Fig. 2. In order to predict the orientation of crystal growth, it must be sufficient to compare the adsorption energy between cubic growth and 45^∘^ growth. The adsorption energy was evaluated with total energy (Etot) of oxide cluster, substrate surface and supercell consisting of the cluster and surface. For low cost computing, the simulation can exclude optimization of substrate surface and cluster migration. Computing cost can be tremendously reduced without those optimizations by using molecular dynamics in the supercell. MgO cluster was simply placed on Si(001) with relation of cubic or 45^∘^ arrangements, and the adsorption energy was estimated without the optimization of surface structure nor cluster migration. In this study, we introduce a simple method to evaluate adsorption energy by simple total of energies method (simple ToE method).

MgO films were experimentally deposited on Si substrate by a pulsed laser deposition (PLD) and examined by variety of X-ray diffraction (XRD) methods, ordinal $\theta$-2$\theta$ scan, ${\text {in-plane} \, \theta -2\theta \, \text {scan}}$, $\phi$ scan, reciprocal space mapping and Pseudopowder XRD^5,28^, and epitaxial growth of MgO was verified on the Si surface. Interestingly, construction of lattice constants is reported on MgO film deposited on Si(001), and under low vacuum atmosphere nano cubic MgO grows on Si surface^29^. Density functional theory(DFT) calculation shows the construction can be caused on defect model of MgO structure^28^, and improves the domain mismatch^5^.

**Figure 3:**
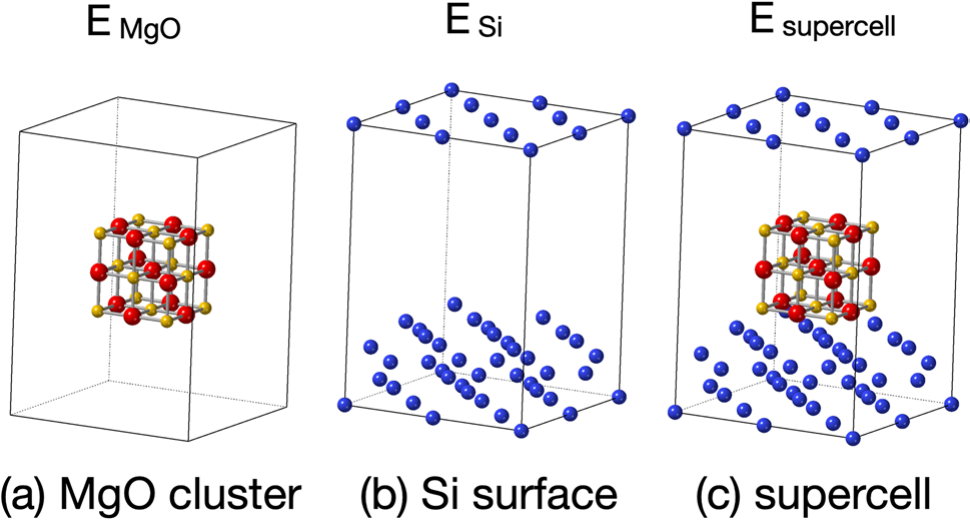
Adsorption energy, $E_{\textrm{ads}}$, was calculated by $E_{\textrm{abs}}$ = $E_{\textrm{supercell}}$ - $E_{\textrm{Si}}$ - $E_{\textrm{MgO}}$.

## Experimental methods

Convention cells of 2$\times$2$\times$2 Si and 1$\times$1$\times$1 MgO cluster were prepared, and vacuum slab of 1 nm, was inserted on 2$\times$2$\times$2 Si surface. Supercell was generated by placing MgO cluster into the vacuum slab on Si surface with varied distances from the Si surface: red planes shown in Fig. 1. The arrangement between MgO and Si were MgO(100) // Si(100) (cubic growth) or MgO(110) // Si(100) (45^∘^ growth). Crystal structure was constructed by using the CrystalMaker X, and converted into appropriate format by using cif2cell^30^.

For calculation of adsorption energy, as shown in Fig. 3, total energies of (a) MgO cluster, (b) Si(001) with vacuum slab and (c) supercell of MgO inserted into vacuum slab were calculated by using the ABINIT code^31^, a project of the Louvain, based on density functional theory. A parallel version of ABINIT was prepared with openmpi and performed on Intel Zeon and Apple M1. The projector augmented wave method (PAW)^32^ was used with the LDA atomic datasets on the ABINIT web site (https://www.abinit.org/psp-tables). The energy difference for self-consistent field was set at 1.0$\times$10^-6^ Hartree (Ha) with energy cut-off of 40 Ha and paw energy cut-off of 60 Ha.

Pulsed laser deposition (PLD) was a versatile method and simple compared to another method like a molecular beam epitaxy^33^ and used for depositing various films^34–36^ including nano particles^37,38^. Film deposition was performed by the PLD using a slower Q-switched YAG laser^39^ with a fourth harmonics of 266 nm at the repetition rate of 2 Hz, and sputtering method was also employed to deposit MgO film on Si(001) substrates. X-ray diffraction was employed to verify the epitaxial growth, in-plane crystal orientation, and film thickness.

## Results and discussion

In spite of large lattice mismatch ($\sim$ 22%), MgO thin film was well known grown epitaxially on Si substrates^1,6,7^. Although 45^∘^ growth (9% mismatch) is preferable than cubic on cubic growth (23%), with a concept of domain mismatch^5,40,41^, cubic on cubic growth can be preferable. In the domain epitaxial growth^42^, *m* unit lattices of the film match with *k* of the Si substrate. The domain coherent strain is defined as with the lattice coherent strain as,

$$2\frac{m a_{\textrm{MgO}} - k a_{\textrm{Si}}}{m a_{\textrm{MgO}} + k a_{\textrm{Si}}},$$

instead of ordinal lattice mismatch as,

$$2\frac{a_{\textrm{MgO}} - a_{\textrm{Si}}}{a_{\textrm{MgO}} + a_{\textrm{Si}}}.$$

Either concept only includes crystallographic relations. Schlom et al. comprehensively investigate the thermodynamic stability of more than 80 binary oxides including MgO, however the thermodynamic stability does not include any factors related with the orientation of crystal growth.

**Figure 4:**
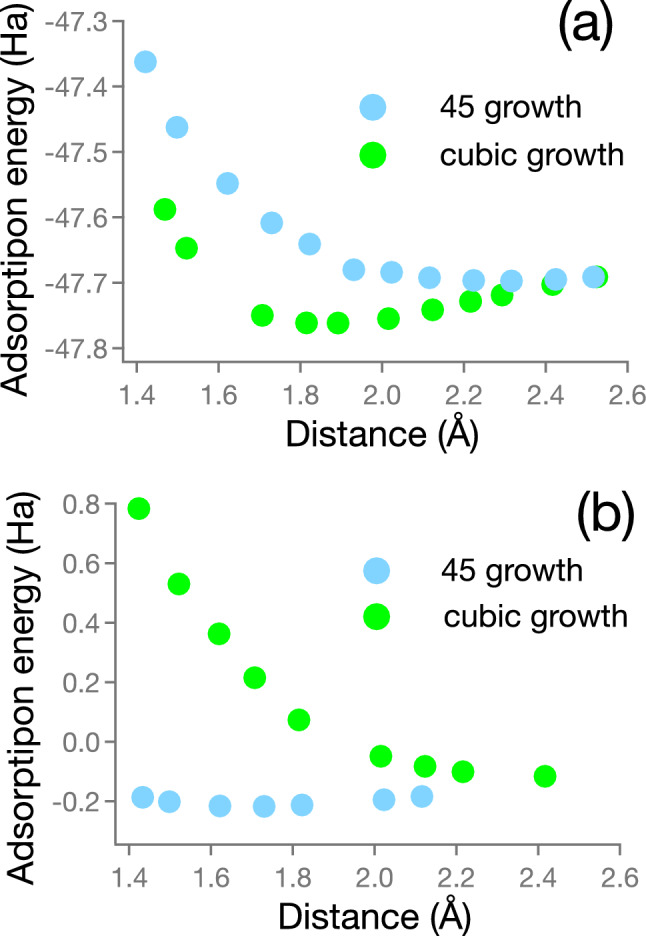
Absorption energy estimated by simple total energy. (**a**) Mg atom and (**b**) O atom placed on Si atom.

In this study, adsorption energy was introduced to evaluate the crystal orientation of epitaxial growth. Adsorption energy was estimated from total energies of MgO cluster, Si surface, and supercell as,

$$E_{\textrm{ads}} = E_{\textrm{supercell}} - E_{\textrm{Si}} - E_{\textrm{MgO}} ,$$

where $E_{\textrm{ads}}$ is an adsorption energy, and $E_{\textrm{supercell}}$, $E_{\textrm{Si}}$ and $E_{\textrm{MgO}}$ shows total energy of supercell, Si surface and MgO cluster, respectively, as shown in Fig. 3. Supercell was constructed by inserting MgO cluster on Si(001) surface with the relation of Mg atom placed on Si atom (Mg-centered) or O atom placed on Si atom (O-centered), and adsorption energy was estimated as shown in Fig. 4a,b. MgO clusters were placed on the Si surface with the distance from 0.14 to 0.25 nm.

The adsorption energy was stable on cubic growth with Mg-centered on Si atom. In the case of O atom placed on Si atom (O-centered), the adsorption energy was quite higher compared to Mg-centered with both cubic and 45^∘^ growth. It might be related to the binding energy of atoms between Mg–Si and O–Si atoms. The cubic growth preferred to 45^∘^ growth agreed with our previous study using the Next-Generation Integrated Supercomputation System at Advanced Fluid Information Research Center, Institute of Fluid Science, Tohoku University. The calculation includes structural optimization and cluster migration together with optimization of supercell structure.

The calculation of the simple ToE method was usually less than 10 or 20 hours for the first time, and couple to several hours for following in chain the calculations in the multi-dataset mode of abinit code. The abinit code restarts calculation with wavefunction generated by previous calculation for speed up. While the calculations took more than 30 hours by the Supercomputation System at Advanced Fluid Information Research Center, the simple ToE method showed the same results less than half computing time on a Home PC, and dependent on convergence condition, the computing time was reduced by less than quarter compared to the calculation by Supercomputation System at Tohoku University. In oder to evaluate the direction of crystal growth, precise calculation is not required for simulations.

**Figure 5:**
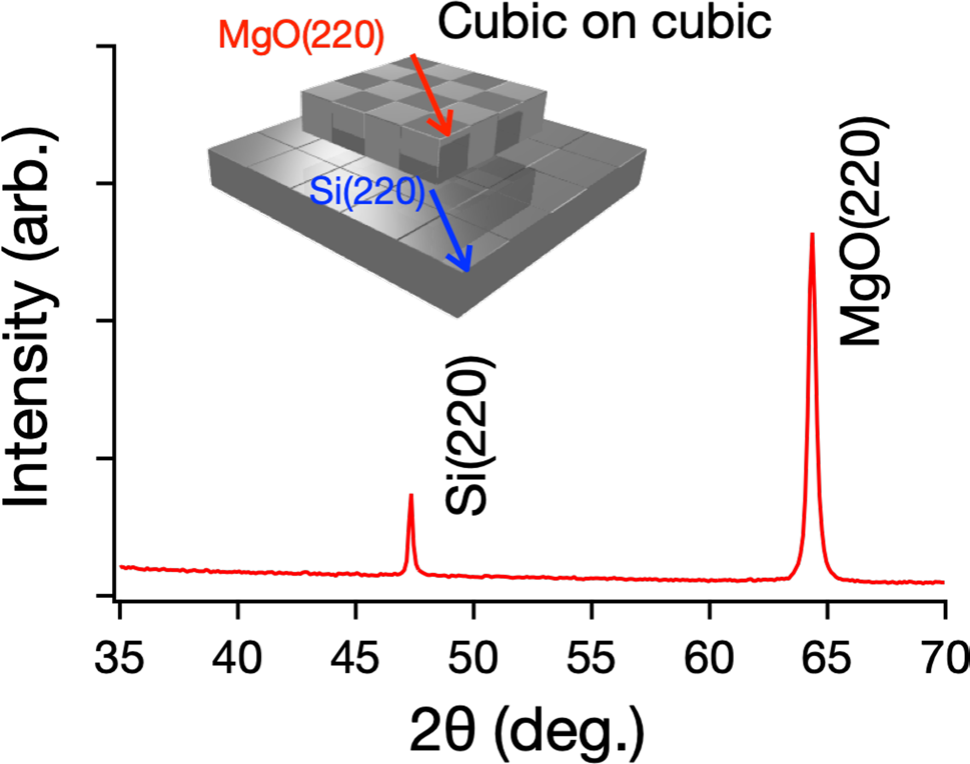
${\text {In-plane}\, \theta -2 \theta \, \text {XRD}}$ of Si(220) peak together with MgO(220) peak, indicating cubic on cubic growth of MgO on Si substrate.

MgO films were experimentally deposited on Si(001) substrates by a PLD system and sputtering methods. Figure 5 shows the ${\text {in-plane} \, \theta -2\theta \, \text {XRD}}$ using Si(220) peak. MgO(220) peak was observed with Si(220) peaks, indicating MgO grew with relation of cubic on cubic growth [MgO(001) // Si(001) and MgO(110) // Si(110)]^5,28^. X-ray reflectivity revealed the film thickness to be $\sim$ 50 nm, ${\text {and surface roughness of} \sim 3\,\text {nm.}}$ The experimental results showed cubic growth, which agreed with the simulation used with the simple ToE method.

For lower computing cost, adsorption energy was evaluated without structural optimization (simple ToE method). Although adsorption energy were estimated to be different values, the adsorption energy showed the same trend on stability of MgO cluster on Si surface. The crystal growth of MgO showed the same direction on Si surface with/without structural optimization. The simple ToE method allows us to evaluate adsorption energy at relatively-low computing cost, and can be performed on home PC.

MgO thin films grew on Si(001) substrate with cubic on cubic growth, and the lattice constant is often contracted^7,43–45^. The first principle theory shows the stability of the contracted structure with Schottky defect model, and the contracted structure result in better domain mismatch^5^. The advantage of cubic on cubic over 45^∘^ rotation growth is supported by (1) thermal stability between MgO and Si^15^, (2) domain epitaxy^5^, (3) the defect model, and (4) crystallographic stability (this work).

## Summary

We proposed the simple ToE method to estimate an adsorption energy for prediction of crystal orientation of epitaxial growth on silicon substrate. The supercell constructed as target cluster inserted into vacuum slab on substrate surface without optimization for surface nor structural optimization. The total energy was simply calculated on the supercell without any optimization, and the absorption energy was estimated as the different energy of the supercells (as shown in Fig. 4). However, the simple method was sufficient for evaluation of the orientation of crystal growth, and computing time was less than half compared to previous report using supercomputer systems. This method is versatile method and can be performed on variety of combination of epitaxial growth.

## Data Availability

The datasets generated during and/or analysed during the current study are available from the corresponding author on reasonable request.
